# Supplementary material for: Variants in the fetal genome near pro-inflammatory cytokine genes on 2q13 associate with gestational duration
Source: Nat Commun. 2019 Sep 2;10:3927. doi: 10.1038/s41467-019-11881-8 (PMC6718389; doi:10.1038/s41467-019-11881-8)
Supplement: Supplementary file 1 — Supplementary Information [file 41467_2019_11881_MOESM1_ESM.pdf]

Supplementary information

Variants in the fetal genome near pro-inflammatory cytokine genes on 2q13 associate with gestational duration

Liu et al.

Table of contents

Supplementary Figures

**Supplementary Figure 1:**	Study design	**4**
**Supplementary Figure 2:**	Manhattan and quantile-quantile plots	**5**
**Supplementary Figure 3:**	Regional association plot of the 2q13 locus for gestational duration and postterm birth	**6**
**Supplementary Figure 4:**	Regional association plot of the 2q13 locus for early preterm birth and preterm birth	**7**
**Supplementary Figure 5:**	Frequency of allele rs7594852-C grouped into bins by gestational age	**8**
**Supplementary Figure 6:**	Regional association plots of the 3q28 and 1p33 loci for early preterm birth	**9**
**Supplementary Figure 7:**	Regional association plots of the 3q28 and 1p33 loci for early preterm birth, conditioning on the lead SNP at each locus	**9**
**Supplementary Figure 8:**	Forest plots of the 3q28 and 1p33 loci for early preterm birth	**10**
**Supplementary Figure 9:**	Quantile-quantile plot of gestational duration associations for 39 SNPs known to be associated with cytokines	**10**
**Supplementary Figure 10:**	Statistical power curves for the discovery stage analyses	**11**
**Supplementary Figure 11:**	Reference-based bimodal gestational duration quantile transformation	**12**

Supplementary Tables

**Supplementary Table 1:**	Statistical power estimates for the replication stage analyses	**13**
**Supplementary Table 2:**	Association results for the two lead SNPs for early preterm birth	**14**
**Supplementary Table 3:**	Replication results for early preterm birth based on trios	**15**
**Supplementary Table 4:**	Association between rs7594852 and gene expression in placenta	**16**
**Supplementary Table 5:**	Association between rs7594852 and levels of 10 biomarkers in blood from newborns	**16**
**Supplementary Table 6:**	Genomic inflation factors for each phenotype in each study	**17**

Supplementary Methods 18

Supplementary Notes

Supplementary Note 1: Early Growth Genetics (EGG) Consortium members **20**

Supplementary Note 2: Collaborators from the iPSYCH-BROAD Working Group **26**

Supplementary Note 3: Study acknowledgements **27**

Supplementary Note 4: Study funding statements **29**

Supplementary Figures

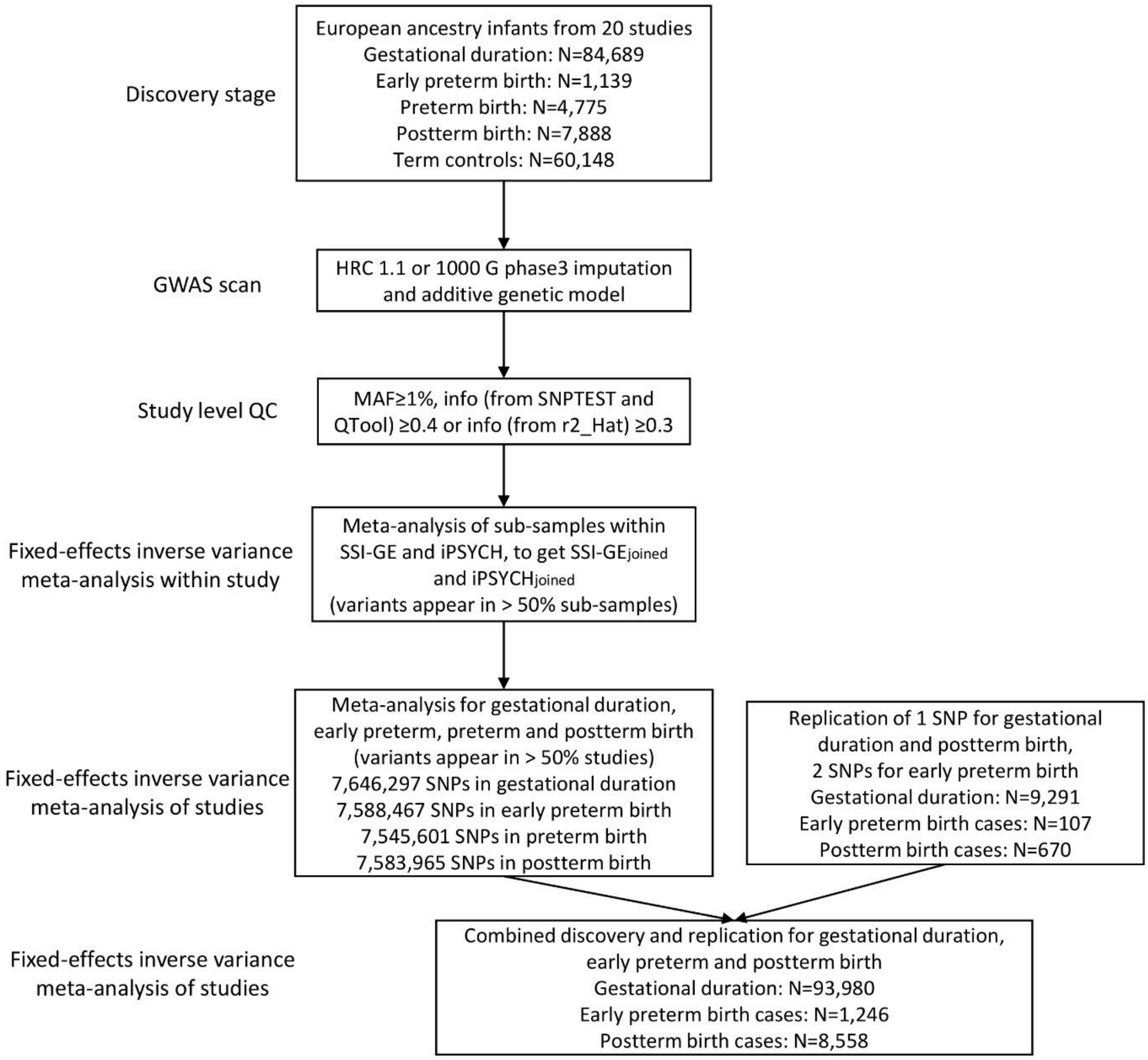

Supplementary Figure 1. Study design.

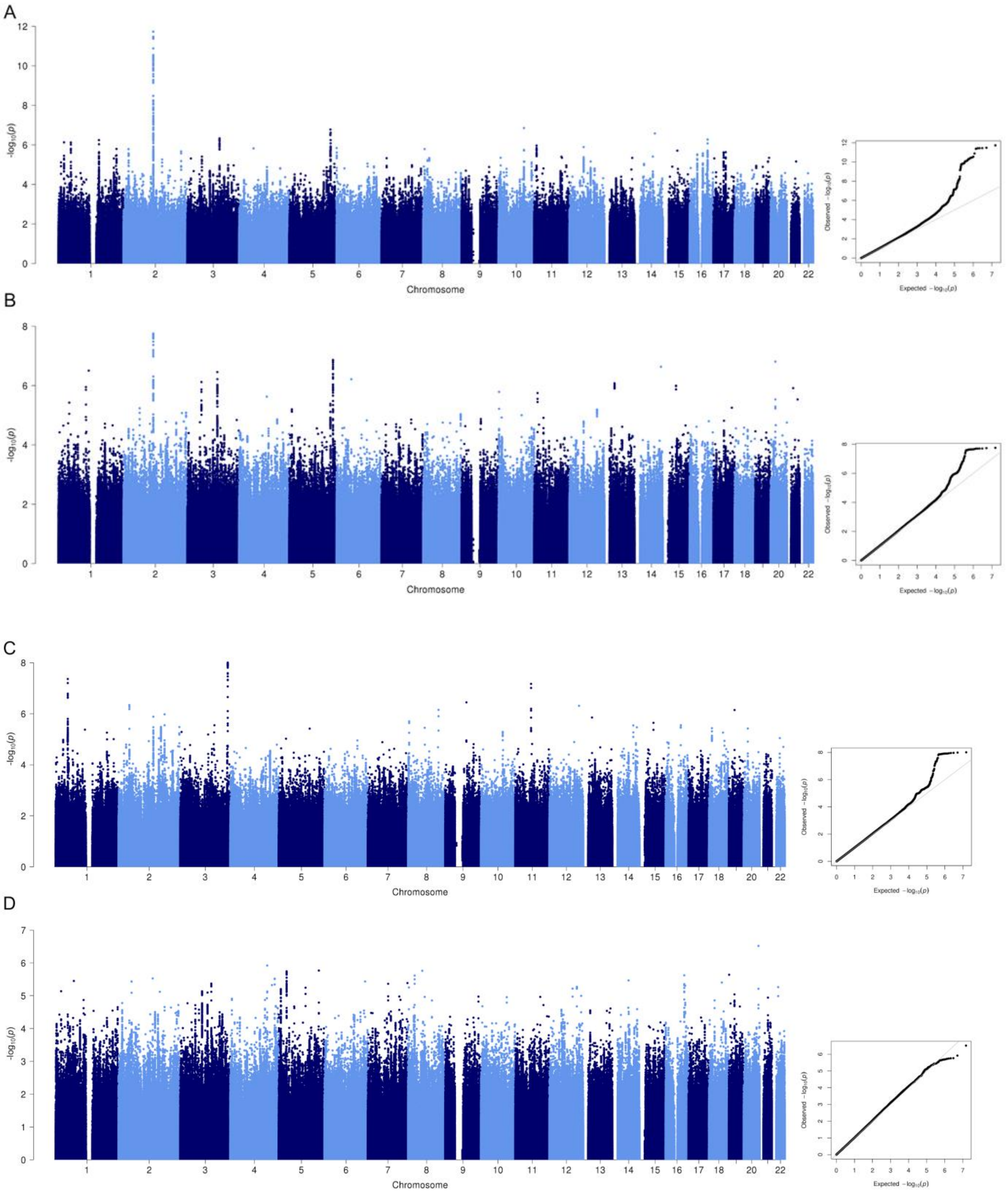

Supplementary Figure 2. Manhattan plots of −log10 *P* values across the chromosomes (left panel) and corresponding quantile-quantile plot of observed versus expected –log10 *P* values (right panel). (**A**) gestational duration, (**B**) postterm birth, (**C**) early preterm birth and (**D**) preterm birth.

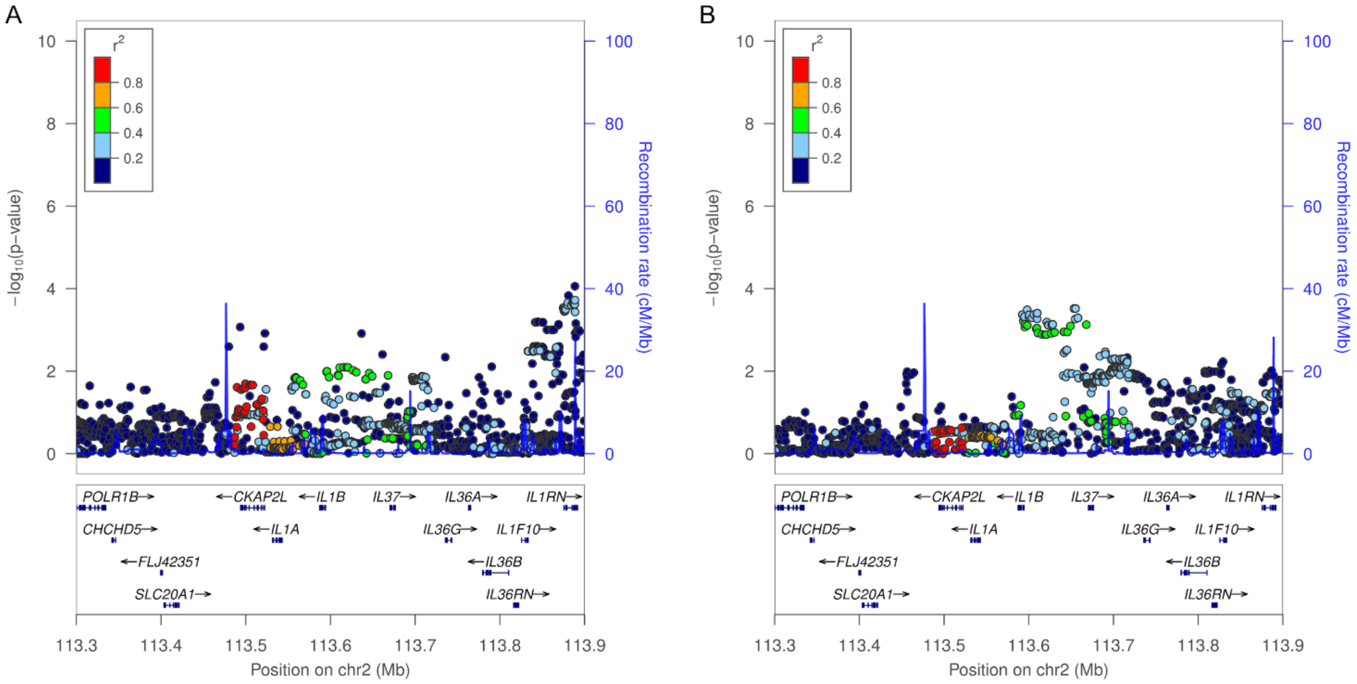

Supplementary Figure 3. Regional association plots of the 2q13 locus, conditioning on rs7594852, for (**A**) gestational duration and (**B**) postterm birth. SNP position is shown on the x-axis and -log10 *P* value on the left y-axis. The conditional analyses were based on 51,357 samples from the iPSYCH study. The SNPs are colored to reflect their linkage disequilibrium with the lead SNP rs7594852 (based on pairwise r^2 values from the DNBC). Estimated recombination rates are from HapMap (right y-axis).

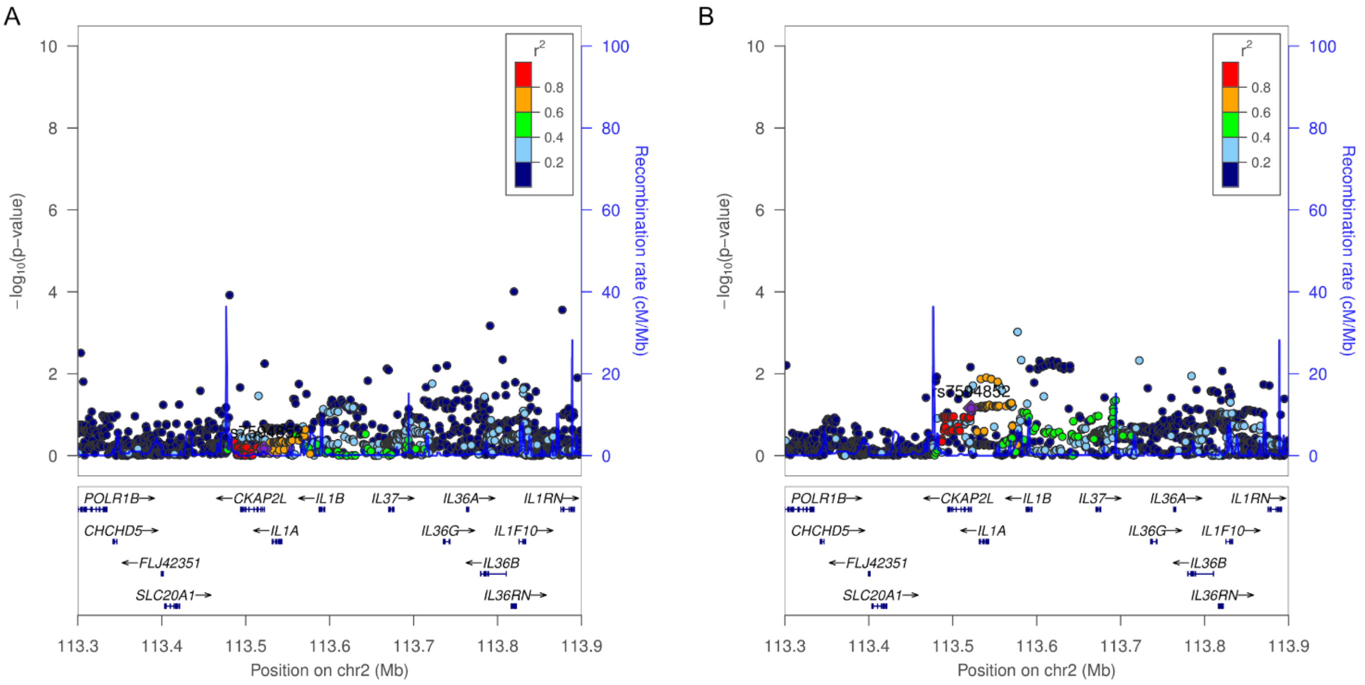

Supplementary Figure 4. Regional association plots of the 2q13 locus for (**A**) early preterm birth, and (**B**) preterm birth. SNP position is shown on the x-axis and -log10 *P* value on the left y-axis. The SNPs are colored to reflect their linkage disequilibrium with the lead SNP for gestational duration, rs7594852 (based on pairwise r^2 values from the DNBC). Estimated recombination rates are from HapMap (right y-axis).

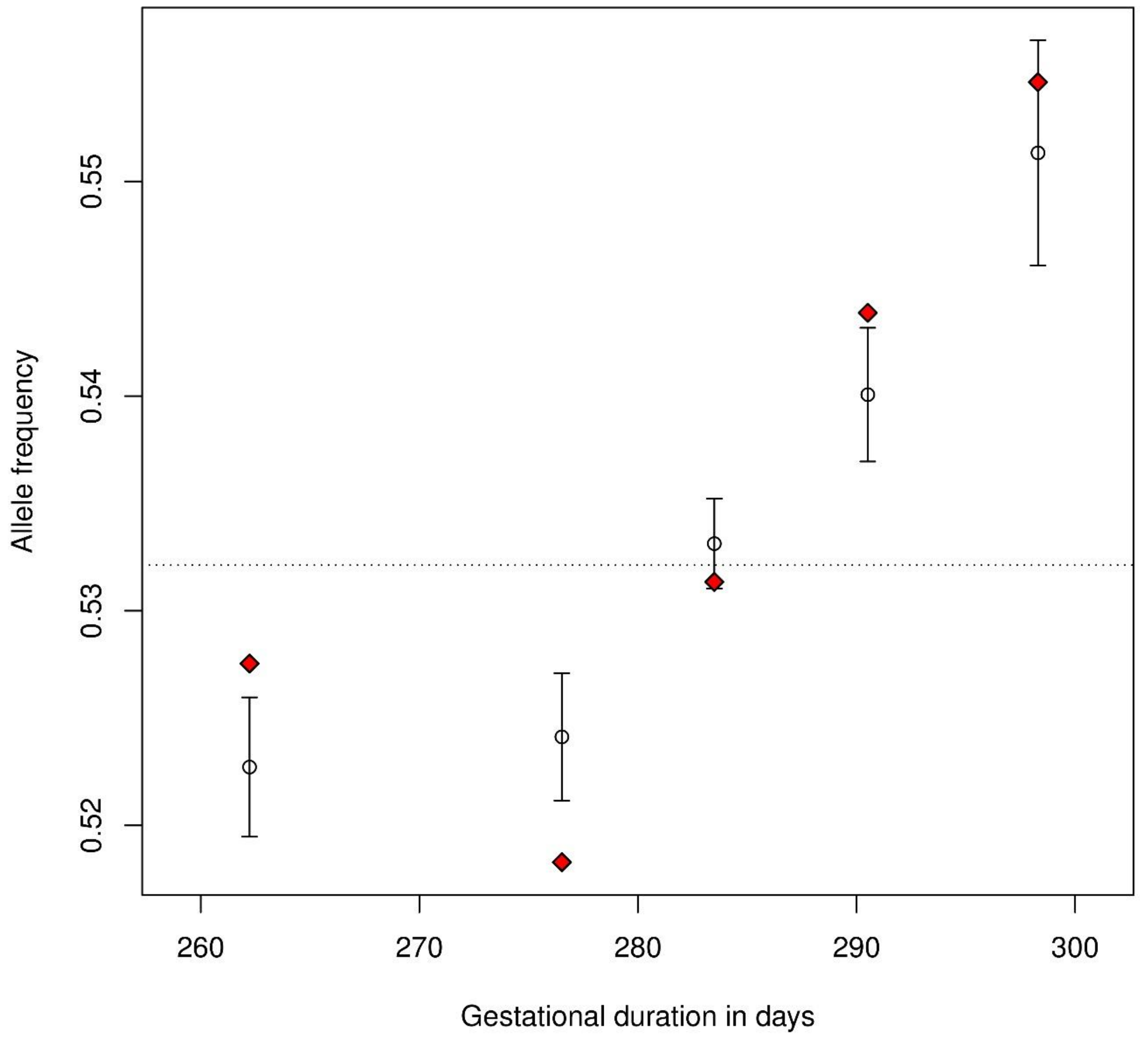

Supplementary Figure 5. Frequency of allele rs7594852-C in 51,357 samples from the iPSYCH study grouped into bins by gestational age. The samples were divided into 5 groups by gestational duration, each red diamond represents a group, the location along the *x*-axis is the mean gestational age of the group, while the *y*-axis is the frequency of allele rs7594852-C in the group. Each circle represents the corresponding bootstrap mean allele frequency and the error-bars are given as the bootstrap standard deviation. The dashed line shows the overall mean allele frequency. Source data are provided as a Source Data file.

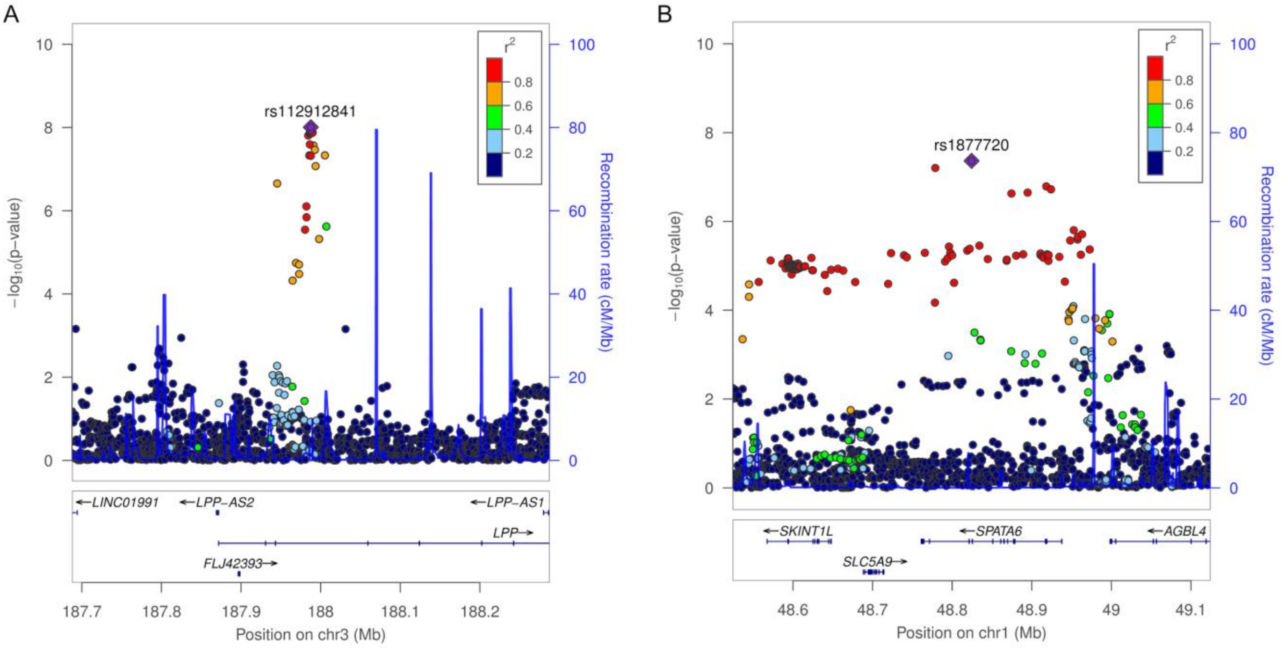

Supplementary Figure 6. Regional association plots of the 3q28 and 1p33 loci for early preterm birth. The lead SNPs, rs112912841 (**A**) and rs1877720 (**B**) are both represented by a purple diamond, and the other SNPs are colored to reflect their LD with the lead SNP (based on pairwise r^2 values from the DNBC cohort). Estimated recombination rates are from HapMap (right *y*-axis).

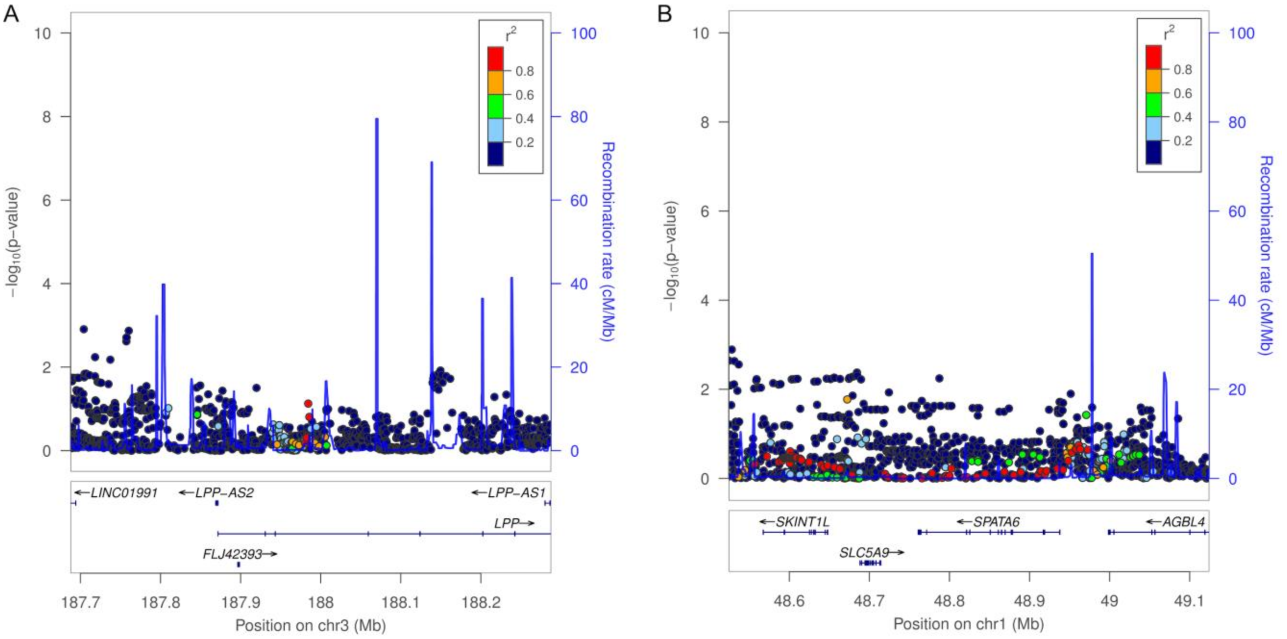

Supplementary Figure 7. Regional association plots of the 3q28 and 1p33 loci for early preterm birth, conditioning on the lead SNP at each locus, rs112912841 (**A**), and rs1877720 (**B**). Conditional analyses were conducted based on 452 iPSYCH early preterm cases and 38,238 controls. The SNPs are colored to reflect their linkage disequilibrium (LD) with the lead SNP (based on pairwise r^2 values from the DNBC cohort).

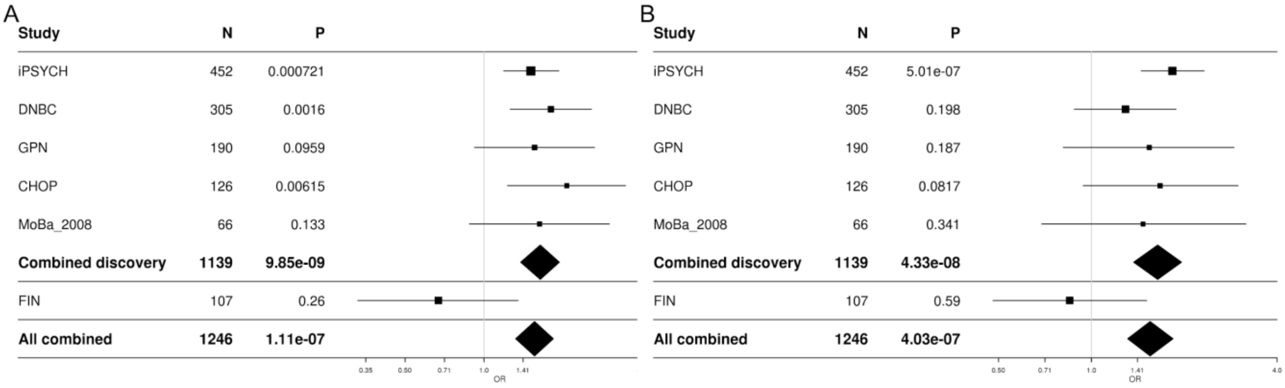

Supplementary Figure 8. Forest plots showing associations between rs112912841 (**A**), rs1877720 (**B**) and early preterm birth in contributing cohorts. The plots show odd ratio estimates with 95% confidence intervals. Source data are provided as a Source Data file.

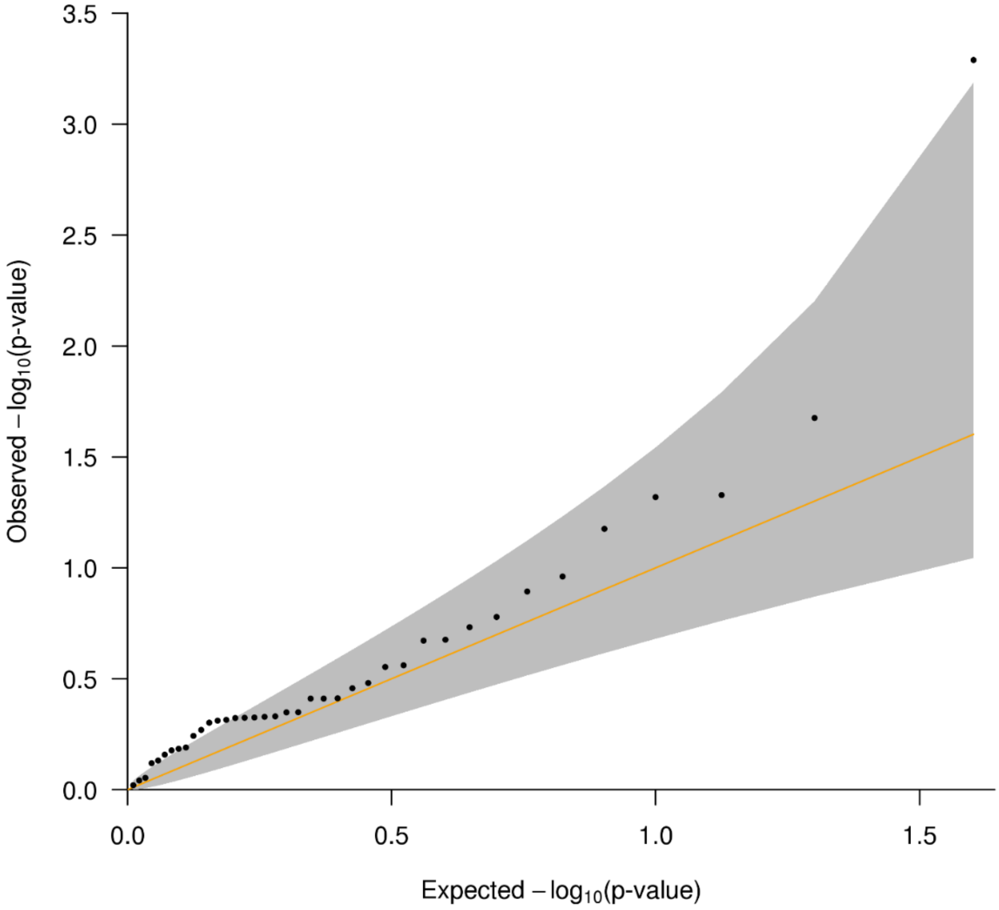

Supplementary Figure 9. Quantile-quantile plot of gestational duration associations for 39 SNPs, which were known to be associated with cytokines. To be considered already known, the association had to be reported in the GWAS catalog with $P < 5 \times 10^{-8}$. Observed versus expected –log10 *P* values are plotted for all SNPs and the orange line represents expected –log10 *P* values under the null distribution. The gray area defines the 95% concentration bands, which are an approximation to the 95% confidence intervals around the expected line. Source data are provided as a Source Data file.

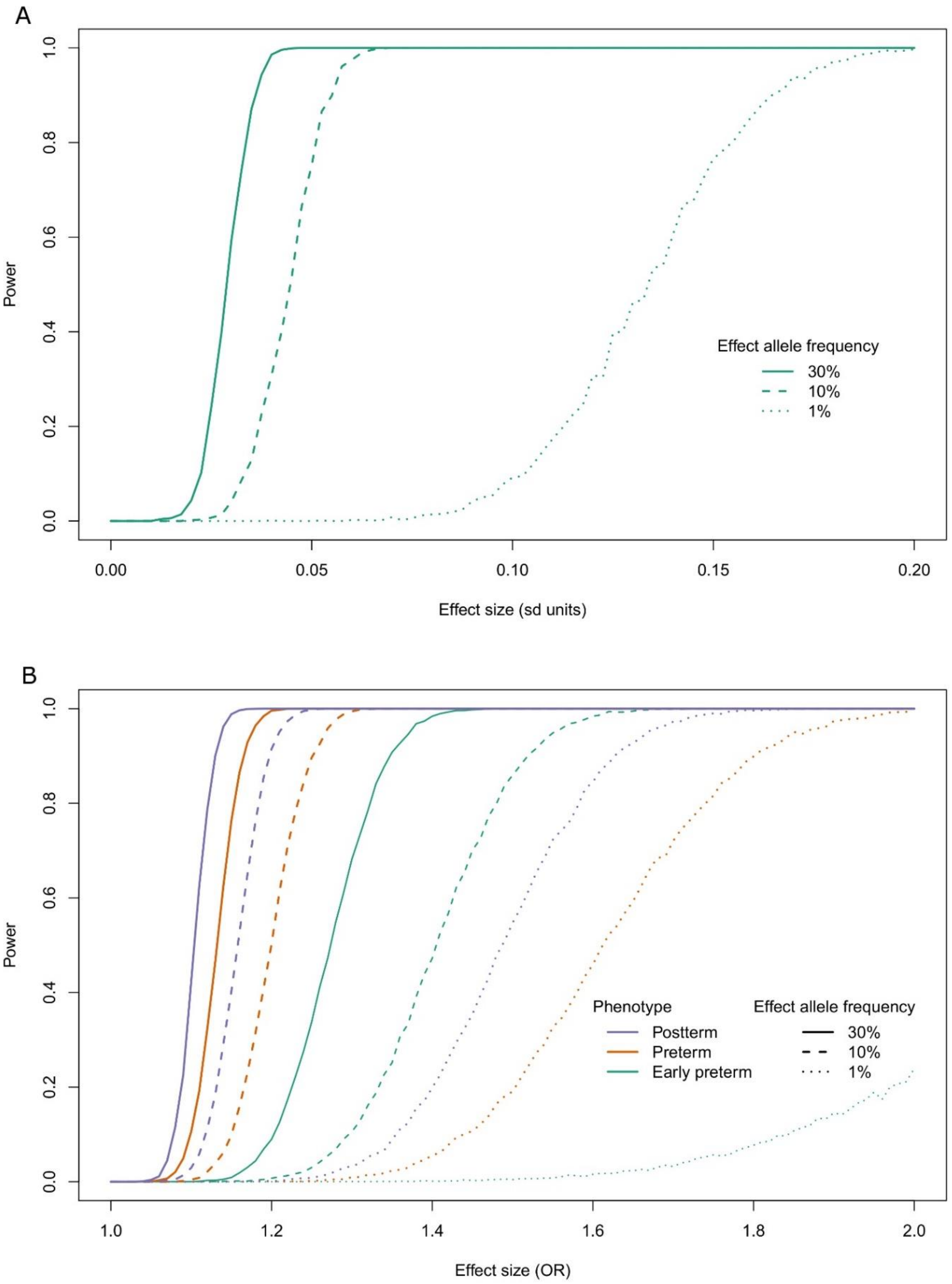

Supplementary Figure 10. Statistical power to detect associated variants in the discovery stage for (**A**) gestational duration and (**B**) postterm birth, preterm birth, and early preterm birth. The power is calculated assuming a significance level of 5×10^{-8}, and population incidences of 1% for early preterm birth and 5% for preterm and postterm birth, respectively. Source data are provided as a Source Data file.

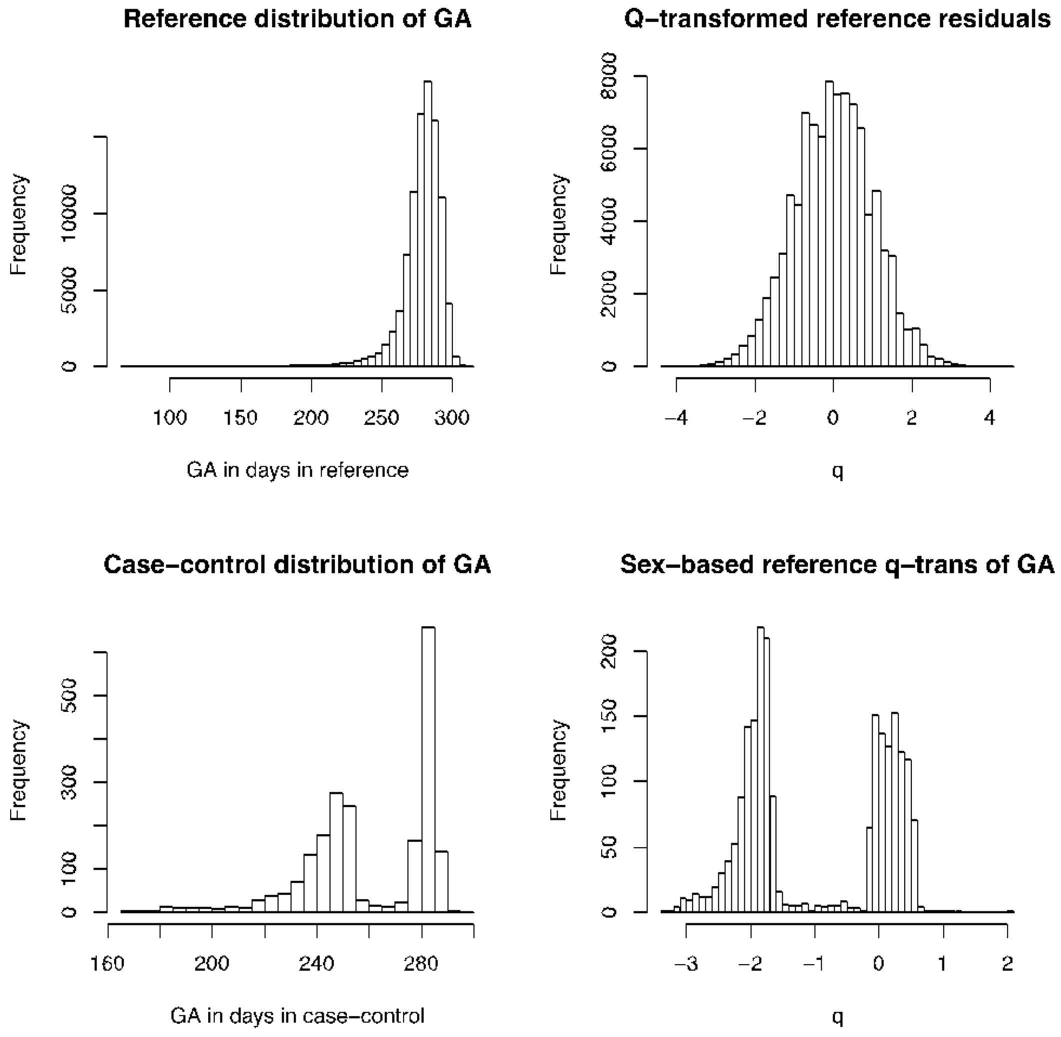

Supplementary Figure 11. Reference-based bimodal gestational duration quantile transformation. Source data are provided as a Source Data file.

Supplementary Tables

Supplementary Table 1. Power calculations for replication stage analyses.

Phenotype	Chromosome Position (bp) SNP (effect/alternate allele)	Population effect allele frequency	Number		Beta/OR	Power
			Cases	Controls		
Gestational duration	2 113521754 rs7594852 (C/T)	0.53		9291	0.034	>99%
Postterm birth	2 113521754 rs7594852 (C/T)	0.53	670	5626	1.1	40%
Early preterm birth	3 187987683 rs112912841 (G/A)	0.061	107	865	1.64	50%
	1 48824407 rs1877720 (T/C)	0.076	107	865	1.64	57%

For the quantitative trait of quantile transformed gestational duration the sample size is given in the "Controls" column, while the effect size Beta is in units of standard deviation. For the dichotomous traits postterm and early preterm birth, odds ratio (OR) estimates are given. The power is calculated assuming a significance level of 0.05, and population incidences of 1% and 5% for early preterm birth and postterm birth, respectively.

Supplementary Table 2. Discovery, replication and combined results for the two lead SNPs in the early preterm birth analysis. Effect size is given as odds ratio (OR) estimates assuming an additive genetic effect; CI, confidence interval; I^2, heterogeneity estimate; P_{het}, *P* value from the *Cochran Q* test of heterogeneity.

Chromosome Position (bp) SNP (effect/ alternate allele)	Sample sets	Effect allele frequency		Number		OR (95% CI)	*P*	I^2 (95% CI)	P_{het}
		cases	controls	cases	controls				
1 48824407 rs1877720 (T/C)	Combined discovery	0.074	0.046	1139	60148	1.64 (1.37–1.96)	4.33×10^{-8}	0 (0.0–67.9)	0.65
	FIN	0.065	0.076	107	865	0.85 (0.48–1.51)	0.59		
	All combined			1246	61013	1.55 (1.31–1.84)	4.03×10^{-7}	78.2 (0.0–99.9)	0.032
3 187987683 rs112912841 (G/A)	Combined discovery	0.093	0.061	1139	60148	1.64 (1.38–1.94)	9.85×10^{-9}	0 (0.0–63.7)	0.83
	FIN	0.042	0.061	107	865	0.67 (0.33–1.35)	0.26		
	All combined			1246	61013	1.56 (1.32–1.84)	1.11×10^{-7}	83.2 (15.7–99.9)	0.015

Supplementary Table 3. Replication results based on mother-father-child trios from Iowa for the two lead SNPs in the early preterm birth discovery analysis. Effect size is given as odds ratio from the transmission disequilibrium test (TDT).

Chromosome Position (bp) SNP (effect/ alternate allele)	Effect allele frequency	Number of informative families	Transmitted minor allele count	Untransmitted allele count	TDT odds ratio	P
1<br>48824407<br>rs1877720 (T/C)	0.041	41	18	24	0.75	0.35
3<br>187987177<br>rs2306375 (A/C)*	0.075	54	28	27	1.04	0.89

*rs2306375 was used as a proxy for rs112912841. These SNP are in perfect LD (r^2=1.0, D'=1.0) in the European populations of the 1000 Genomes Project.

Supplementary Table 4. Association between rs7594852 (effect/non-effect allele: C/T) and gene expression in placenta. All cis genes (among 118 genes within 500kb from the SNP) where the eQTL association *P* value was below 0.05 are listed.

Transcript ID	Gene	R^2	BETA	SE	*P*
ENSG00000144136	*SLC20A1*	0.13	-0.50	0.13	0.00027
ENSG00000136688	*IL36G*	0.095	-0.44	0.14	0.0017
ENSG00000115008	*IL1A*	0.072	-0.38	0.14	0.0065
ENSG00000180152	*AC079753.4*	0.044	-0.28	0.13	0.033
ENSG00000136695	*IL36RN*	0.040	0.29	0.14	0.043

Supplementary Table 5. Association between rs7594852 (effect/non-effect allele: C/T) and levels of 10 biomarkers measured in peripheral blood taken a few days after birth from 8,138 participants of the iPSYCH study.

Biomarker	BETA	SE	*P*
BDNF	0.0004	0.0082	0.9566
CRP	0.0267	0.0183	0.1445
EPO	0.0108	0.0106	0.3124
IgA	0.0069	0.0095	0.4712
IL-18	0.0011	0.0075	0.8876
IL8	-0.0136	0.0087	0.1175
MCP1	0.0047	0.0052	0.3644
S100B	-0.0110	0.0148	0.4571
TARC	-0.0087	0.0109	0.423
VEGFA	-0.0021	0.0067	0.7559

Supplementary Table 6. Genomic inflation factors for each phenotype in each study.

Study	Early preterm birth	Preterm birth	Postterm birth	Gestational duration
ALSPAC	NA	1.003	1.014	1.006
CHOP	1.012	1.009	NA	1.017
COPSAC2000	NA	NA	1.012	1.052
COPSAC2010	NA	NA	NA	1.010
COPSAC_REGISTRY	NA	NA	1.004	0.998
DNBC	1.014	0.998	NA	1.010
EFSOCH	NA	NA	NA	1.003
GenR	NA	NA	1.007	0.998
HAPO	NA	NA	NA	0.994
INMA	NA	NA	NA	1.000
MoBa_2008	0.969	0.981	NA	0.992
NFBC1966	NA	1.007	1.005	1.009
NFBC1986	NA	1.011	NA	1.007
Raine Study	NA	NA	NA	0.994
SSI-GE	NA	NA	0.975	1.007
STRIP	NA	NA	NA	1.011
1958BC (DIL-T1DGC)	NA	0.986	0.996	0.991
1958BC (WTCCC)	NA	0.981	1.014	1.002
iPSYCH	1.007	1.007	1.026	1.044
GPN	0.989	0.988	NA	NA

Supplementary Methods

Semi-parametric bootstrap of binned allele frequencies to test for non-linearity.

As described in the main text, no association was seen at the 2q13 locus in case-control analyses of early preterm birth or preterm birth. This may suggest that other mechanisms could be playing a greater role in causing early parturition before the mechanisms mediating the effect of the locus get their chance to influence the phenotype. To further investigate this question, we binned the 51,357 births from the largest contributing study (iPSYCH) in five groups by gestational duration. We then estimated the frequency of the rs7594852-C allele in each group and in the whole sample.

In the overall meta-analysis, each additional fetal rs7594852-C allele was associated with increased gestational duration (**Table 1**). The frequency of the rs7594852-C allele in the group with the shortest gestational duration was only slightly lower than the frequency in the whole sample (**Supplementary Figure 5**). The lowest allele frequency (0.518) was seen in the second group, representing a mean gestational duration of 276.5 days. The allele frequency then gradually increased in the next groups with the highest frequency (0.555) observed for the group representing the longest gestational duration (mean of 298.3 days) (**Supplementary Figure 5**).

To investigate if this pattern in allele frequencies represents a statistically significant deviation from what is expected under the hypothesis that the strength of the association is independent of gestational duration, we carried out semi-parametric bootstrapping under the assumption H_0: "the variant contributes equally to higher gestational duration no matter when the child is born". We chose a semi-parametric bootstrap approach to avoid assuming normal-distributed residuals in the untransformed distribution of gestational duration.

Our test statistic is based on bootstrapping allele frequencies in the five bins under H_0. If the variant influences gestational duration less in the early part of the distribution, then the observed allele frequency $f1$ in the first bin will be closer to the overall frequency than expected under H_0, while the allele frequency in the second bin ($f2$) will be lower than expected under H_0 and in the fifth bin ($f5$) the allele frequency will be higher than expected under H_0.

Semi-parametric bootstrap was performed based on imputed genotype dosages and gestational duration in days from the 51,357 iPSYCH samples.

First, observed allele frequencies ($f1$, ..., $f5$) in the gestational duration intervals (0,273), [273,280), [280,287), [287,294) and [294,315) were calculated, as stated above and in the main text.

Next, expected gestational duration conditional on genotype was estimated using least squares regression assuming a linear relationship in the whole range of gestational duration:

$$E[\,y\mid g\,] = a + b * g \tag{1}$$

Empirical residuals were then extracted based on the expected gestational duration given the genotype:

$$r = y - \hat{a} - \hat{b} * g \tag{2}$$

Given the genotype g, gestational duration was now bootstrapped under the null hypothesis with resampling of the empirical residuals:

$$y_{boot} = \hat{a} + \hat{b} * g + r_{boot} \tag{3}$$

Based on the bootstrapped gestational duration, bootstrapped allele frequencies ($f1_{\text{boot}}$, ..., $f5_{\text{boot}}$) in the intervals (0,273), [273,280), [280,287), [287,294) and [294,315) were estimated. The bootstrap procedure was repeated 10,000 times.

The non-linearity *P* value was calculated as:

$$P = \frac{1}{10000} \sum_{boot=1}^{10000} 1(f1_{boot} > f1) * 1(f2_{boot} < f2) * 1(f5_{boot} > f5) \quad (4)$$

The expected allele frequency under H_0 in interval *i* was estimated as the mean of the bootstrapped allele-frequencies fi_{boot}, and the standard deviations of the respective bootstrapped distributions were likewise calculated.

Based on 10,000 joint bootstrap distributions of gestational age and genotype, we estimate that the probability under H_0 of observing more deviating allele frequencies is $P = 0.0013$. The expected binned allele frequencies under H_0 obtained from the bootstrap procedure are illustrated in **Supplementary Figure 5**.

Reference-based bimodal gestational duration quantile transformation

The DNBC and MoBa_2008 cohorts represent case-control studies of preterm birth, which means that the distribution of gestational duration is bimodal for these studies. In these two cohorts, we transformed gestational duration to be on the same scale as the population-based cohorts. For that purpose, we used gestational duration and sex of a representative (random) population based sample from Denmark and Norway, respectively, as reference data.

In the population based reference sample, we first regressed gestational duration on infant sex. We stored the coefficients (intercept *a_ref* and effect of sex *b_ref*) and calculated the residuals *x_ref* in the population based reference sample (*x_ref* = gestational duration - (*a_ref* + *b_ref* * sex)). We mapped the reference residuals, *x_ref,* to quantile transformed residuals, *q_ref*, using a rank-based inverse normal transformation and we stored the pairs (*x_ref*, *q_ref*) of reference residual and transformed reference residual. Having established a transformation from gestational duration to quantile transformed residuals in the population based reference sample, we calculated the equivalent transformation of the gestational duration in the corresponding case-control studies (DNBC and MoBa_2008, respectively). To do so, we first calculated pseudo residuals, *x_cc*, from gestational duration in the case-control study based on the stored coefficients from the reference sample (*x_cc* = gestational duration - (*a_ref* + *b_ref**sex)). Based on the pseudo residual, *x_cc*, in the case-control study, a transformed pseudo residual, *q_cc*, was obtained by linear interpolation from the pairs (*x_ref, q_ref*) of residuals and transformed residuals from the reference population sample. Finally, the transformed pseudo residuals were used for association testing in the DNBC and MoBa_2008 cohorts. The reference-based quantile transformation is illustrated in **Supplementary Figure 11**.

Supplementary Notes

Supplementary Note 1. EGG Membership

Full list of EGG Consortium members (as of July 2019), listed in alphabetical order.

Linda S Adair[1], Emma Ahlqvist[2], Tarunveer S Ahluwalia[3,4,5], Peter Almgren[2], Wei Ang[6], Mustafa Atalay[7], Robin N Beaumont[8], Jacques S Beckmann[9], Tom Bond[10], Klaus Bønnelykke[3], Dorret I Boomsma[11,12,13,14], Judith B Borja[15,16], Jonathan P Bradfield[17,18], Mariona Bustamante[19,20,21], Pimphen Charoen[10,22], Lachlan Coin[23], Cyrus Cooper[24], Diana L Cousminer[25,26], John A Curtin[27], Adnan Custovic[28], Shikta Das[29], Felix R Day[30], N Maneka De Silva[10], George V Dedoussis[31], Paul Elliott[10], Johan G Eriksson[32,33,34], David M Evans[35,36,37], João Fadista[38], Bjarke Feenstra[38], Janine F Felix[39,40,41], Timothy M Frayling[8], Rachel M Freathy[8,35], Romy Gaillard[40], Frank Geller[38], Vincente Gilsanz[42], Struan FA Grant[17,25,26,43], Niels Grarup[4], Leif Groop[2,44], Monica Guxens[19,20,21], Dexter Hadley[45], Hakon Hakonarson[17,25,43], Torben Hansen[4], Andrew T Hattersley[8,46], M Geoffrey Hayes[47], Johannes Hebebrand[48], Joachim Heinrich[49,50], Øyvind Helgeland[51,52,53], Tine B Henriksen[54], Anke Hinney[48], Joel N Hirschhorn[55,56,57], Marie-France Hivert[58,59,60], Berthold Hocher[61,62], John W Holloway[63], Momoko Horikoshi[64,65,66], Jouke-Jan Hottenga[11,12,14], Elina Hyppönen[67,68,69], Bo Jacobsson[53,70], Vincent WV Jaddoe[39,40,41], Marjo-Riitta Järvelin[10,71,72,73,74], Stefan Johansson[51,75], Heidi J Kalkwarf[76], Antje Körner[77,78], Sailesh Kotecha[79], Eskil Kreiner-Møller[3,80], Zoltán Kutalik[81,82], Timo A Lakka[7,83,84], Joan M Lappe[85], Debbie A Lawlor[35,36,86], Terho Lehtimäki[87,88], Alexandra M Lewin[10], Cecilia M Lindgren[64,89,90], Virpi Lindi[7], Allan Linneberg[91,92], Xueping Liu[38], Jun Liu[40], William L Lowe Jr[47], Ronald CW Ma[93,94,95], Reedik Mägi[96], Per Magnus[97], Anubha Mahajan[64,65], Mark I McCarthy[64,65,98], Nina S McCarthy[99], Mads Melbye[38,100], Karen L Mohlke[101], Claire Monnereau[39,40,41], Dennis O Mook-Kanamori[102,103], Camilla S Morgen[104], Andrew P Morris[64,96,105], Louis Muglia[106,107,108], Jeffrey C Murray[109], Ronny Myhre[110], Pål R Njølstad[51,52], Ellen A Nohr[111], Ioanna Ntalla[112], Paul O'Reilly[113], Sharon E Oberfield[114], Emily Oken[115], Ken K Ong[30,116], Kalliope Panoutsopoulou[117], Oluf Pedersen[4], Craig E Pennell[118], John RB Perry[30], Niina Pitkänen[119], Beate St Pourcain[35,120], Christine Power[69], Rashmi B Prasad[2], Inga Prokopenko[64,121], Olli T Raitakari[119,122], Rebecca M Reynolds[123], Rebecca C Richmond[35,36], Alina Rodriguez[10,124], Rany Salem[56,125,126,127], Seang-Mei Saw[128,129], Theresia M Schnurr[4], Sylvain Sebert[10,71,73,130], John A Shepherd[131], Angela Simpson[27], Line Skotte[38], Thorkild IA Sørensen[4,35,104], Marie Standl[49], Eric AP Steegers[132], David P Strachan[133], Jordi Sunyer[19,20,21,134], Michelle Taylor[35,36], Yik-Ying Teo[128,135,136], Elisabeth Thiering[49,137], Nicholas J Timpson[35,36], Jessica Tyrrell[8,138], André G Uitterlinden[39,40,139], Cornelia M van Duijn[40], Suzanne Vogelezang[39,40,41], Tanja GM Vrijkotte[140], Carol A Wang[118], Nicole M Warrington[37], William J Watkins[79], H-Erich Wichmann[49,141,142], Elisabeth E Widén[44], Gonneke Willemsen[11,12,14], James F Wilson[143,144], Hanieh Yaghootkar[8], Mohammad Hadi Zafarmand[140,145], Eleftheria Zeggini[117,146], Babette S Zemel[43,147], Ge Zhang[106,107,108]

1. Department of Nutrition, University of North Carolina, Chapel Hill, NC 27599, USA.
2. Department of Clinical Sciences, Diabetes and Endocrinology, Lund University Diabetes Centre, Malmö, SE-205 02, Sweden.
3. COPSAC, Copenhagen Prospective Studies on Asthma in Childhood, Herlev and Gentofte Hospital, University of Copenhagen, Copenhagen, 2900 Hellerup, Denmark.
4. Novo Nordisk Foundation Center for Basic Metabolic Research, Faculty of Health and Medical Sciences, University of Copenhagen, Copenhagen, DK-2200, Denmark.
5. Steno Diabetes Center Copenhagen, Gentofte, 2820, Denmark.
6. Division of Obstetrics and Gynaecology, The University of Western Australia, Crawley, WA, 6009, Australia.
7. Institute of Biomedicine, School of Medicine, University of Eastern Finland, Kuopio, 70211, Finland.
8. Institute of Biomedical and Clinical Science, College of Medicine and Health, University of Exeter, Royal Devon and Exeter Hospital, Exeter, EX2 5DW, UK.

9. University of Lausanne, Lausanne, CH-1015, Switzerland.
10. Department of Epidemiology and Biostatistics, MRC-PHE Centre for Environment & Health, School of Public Health, Imperial College London, London, W2 1PG, UK.
11. Department of Biological Psychology, Vrije Universiteit Amsterdam, Amsterdam, 1081 BT, The Netherlands.
12. Amsterdam Public Health, Amsterdam, The Netherlands.
13. Amsterdam Reproduction and Development, Amsterdam, The Netherlands.
14. Netherlands Twin Register, Department of Biological Psychology, VU University, Amsterdam, 1081 HV, The Netherlands.
15. USC-Office of Population Studies Foundation, Inc., University of San Carlos, Cebu City, 6000, Philippines.
16. Department of Nutrition and Dietetics, University of San Carlos, Cebu City, 6000, Philippines.
17. Center for Applied Genomics, The Children's Hospital of Philadelphia, Philadelphia, PA 19104, USA.
18. Quantinuum Research LLC, San Diego, CA, 92101, USA.
19. ISGlobal, Institute for Global Health, Barcelona, 08003, Spain.
20. Universitat Pompeu Fabra (UPF), Barcelona, 08003, Spain.
21. CIBER de Epidemiología y Salud Pública (CIBERESP), Madrid, 28029, Spain.
22. Department of Tropical Hygiene, Faculty of Tropical Medicine, Mahidol University, Bangkok, 10400, Thailand.
23. Institute for Molecular Bioscience, University of Queensland, QLD, Australia.
24. Medical Research Council Lifecourse Epidemiology Unit, University of Southampton, Southampton, SO17 1BJ, UK.
25. Division of Human Genetics, The Children's Hospital of Philadelphia, Philadelphia, PA 19104, USA.
26. Department of Genetics, Perelman School of Medicine, University of Pennsylvania, Philadelphia, 19104, USA.
27. Division of Infection Immunity and Respiratory Medicine, School of Biological Sciences, The University of Manchester, Manchester Academic Health Science Centre, and Manchester University NHS Foundation Trust, Manchester, M13 9NT, UK.
28. Department of Paediatrics, Imperial College London, London, SW7 2AZ, UK.
29. Medical Research Council Unit for Lifelong Health and Ageing at UCL, Institute of Cardiovascular sciences, UCL, London, WC1B 5JU, UK.
30. MRC Epidemiology Unit, University of Cambridge School of Clinical Medicine, Cambridge, CB2 0QQ, UK.
31. Department of Nutrition and Dietetics, School of Health Science and Education, Harokopio University, Athens, 17671, Greece.
32. National Institute for Health and Welfare, Helsinki, 00271, Finland.
33. Department of General Practice and Primary Health Care, University of Helsinki and Helsinki University Hospital, Helsinki, 00014, Finland.
34. Folkhälsan Research Center, Helsinki, 00250, Finland.
35. Medical Research Council Integrative Epidemiology Unit at the University of Bristol, Bristol, BS8 2BN, UK.
36. Population Health Sciences, Bristol Medical School, University of Bristol, Bristol, BS8 2BN, UK.
37. University of Queensland Diamantina Institute, Translational Research Institute, Brisbane, QLD, 4072, Australia.
38. Department of Epidemiology Research, Statens Serum Institute, Copenhagen, DK-2300, Denmark.
39. The Generation R Study Group, Erasmus MC, University Medical Center Rotterdam, Rotterdam, 3015 CE, The Netherlands.
40. Department of Epidemiology, Erasmus MC, University Medical Center Rotterdam, Rotterdam, 3015 CE, The Netherlands.
41. Department of Pediatrics, Erasmus MC, University Medical Center Rotterdam, Rotterdam, 3015 CE, The Netherlands.

42. Department of Radiology, Children's Hospital Los Angeles, Los Angeles, CA 90027, USA.
43. Department of Pediatrics, Perelman School of Medicine, University of Pennsylvania, Philadelphia, PA 19104, USA.
44. Institute for Molecular Medicine Finland FIMM, University of Helsinki, Helsinki, Finland.
45. Department of Pediatrics, University of California San Francisco School of Medicine, San Francisco, CA 94143, USA.
46. NIHR Exeter Clinical Research Facility, University of Exeter College of Medicine and Health and Royal Devon and Exeter NHS Foundation Trust, Exeter, EX2 5DW, UK.
47. Department of Medicine, Division of Endocrinology, Metabolism, and Molecular Medicine, Feinberg School of Medicine, Northwestern University, Chicago, IL 60611, USA.
48. Department of Child and Adolescent Psychiatry, Psychosomatics and Psychotherapy, University Hospital Essen, University of Duisburg-Essen, Essen, 45141, Germany.
49. Institute of Epidemiology, Helmholtz Zentrum München - German Research Center for Environmental Health, Neuherberg, Germany.
50. Institute and Outpatient Clinic for Occupational, Social and Environmental Medicine, University Hospital of Ludwig Maximilians University, Munich, Germany.
51. KG Jebsen Center for Diabetes Research, Department of Clinical Science, University of Bergen, Bergen, N-5020, Norway.
52. Department of Pediatrics, Haukeland University Hospital, Bergen, 5021, Norway.
53. Department of Genetics and Bioinformatics, Domain of Health Data and Digitalisation, Norwegian Institute of Public Health, Oslo, N-0473, Norway.
54. Department of Paediatrics and Adolescent Medicine, Aarhus University Hospital, Aarhus N, DK-8200, Denmark.
55. Programs in Metabolism and Medical & Population Genetics, Broad Institute of Harvard and MIT, Cambridge, MA 02142, USA.
56. Department of Genetics, Harvard Medical School, Boston, MA 02115, USA.
57. Division of Endocrinology and Center for Basic and Translational Obesity Research, Boston Children's Hospital, Boston, MA, 02115, USA.
58. Department of Population Medicine, Harvard Pilgrim Health Care Institute, Harvard Medical School, Boston, MA 02215, USA.
59. Diabetes Center, Massachusetts General Hospital, Boston, MA 02114, USA.
60. Department of Medicine, Universite de Sherbrooke, Sherbooke, QC J1K 2R1, Canada.
61. Fifth Department of Medicine, University Medical Centre Mannheim, University of Heidelberg, Heidelberg, Germany.
62. Reproductive and Genetic Hospital of CITIC-Xiangya, Changsha, China.
63. Human Development & Health, Faculty of Medicine, University of Southampton, Southampton, SO16 6YD, UK.
64. Wellcome Centre for Human Genetics, University of Oxford, Oxford, OX3 7BN, UK.
65. Oxford Centre for Diabetes, Endocrinology and Metabolism, University of Oxford, Oxford, OX3 7LE, UK.
66. RIKEN, Centre for Integrative Medical Sciences, Laboratory for Endocrinology, Metabolism and Kidney diseases, Yokohama, Kanagawa, 230-0045, Japan.
67. Australian Centre for Precision Health, University of South Australia Cancer Research Institute, Adelaide, SA, 5001, Australia.
68. South Australian Health and Medical Research Institute, Adelaide, SA, 5001, Australia.
69. Population, Policy and Practice, UCL Great Ormond Street Institute of Child Health, University College London, London, WC1N 1EH, UK.
70. Department of Obstetrics and Gynecology, Sahlgrenska Academy, University of Gothenburg, Diagnosvägen 15, SE-416 85 Gothenburg, Sweden.
71. Biocenter Oulu, University of Oulu, Oulu, 90220, Finland.
72. Unit of Primary Care, Oulu University Hospital, Oulu, 90220, Finland.

73. Center for Life Course Health Research, Faculty of Medicine, University of Oulu, Oulu, FI-90014, Finland.
74. Department of Life Sciences, College of Health and Life Sciences, Brunel University London, Middlesex, UB8 3PH, UK.
75. Department of Medical Genetics, Haukeland University Hospital, Bergen, Norway.
76. Division of Gastroenterology, Hepatology and Nutrition, Cincinnati Children's Hospital Medical Center, Cincinnati, OH 45229, USA.
77. Pediatric Research Center, Department of Women´s & Child Health, University of Leipzig, Leipzig, 04109, Germany.
78. IFB Adiposity Diseases, University of Leipzig, Leipzig, 04109, Germany.
79. Department of Child Health, School of Medicine, Cardiff Univeristy, Cardiff, CF10 3AT, UK.
80. Danish Pediatric Asthma Center, Copenhagen University Hospital, Gentofte, DK-2100, Denmark.
81. Institute of Social and Preventive Medicine, Lausanne University Hospital (CHUV), Lausanne, 1011, Switzerland.
82. Swiss Institute of Bioinformatics, Lausanne, 1015, Switzerland.
83. Department of Clinical Physiology and Nuclear Medicine, Kuopio University Hospital, Kuopio, 70210, Finland.
84. Kuopio Research Institute of Exercise Medicine, Kuopio, 70100, Finland.
85. Division of Endocrinology, Department of Medicine, Creighton University, Omaha, NE 68178, USA.
86. Bristol NIHR Biomedical Research Centre, Bristol, UK.
87. Department of Clinical Chemistry, Fimlab Laboratories, Tampere University Hospital, Tampere, Finland.
88. Department of Clinical Chemistry, Finnish Cardiovascular Research Center - Tampere, Faculty of Medicine and Health Technology, Tampere University, Tampere, 33520, Finland.
89. Li Ka Shing Centre for Health Information and Discovery, The Big Data Institute, University of Oxford, Oxford, OX3 7LF, UK.
90. The Broad Institute of Harvard and MIT, Cambridge, USA.
91. Center for Clinical Research and Prevention, Bispebjerg and Frederiksberg Hospital, The Capital Region, Frederiksberg, 2000, Denmark.
92. Department of Clinical Medicine, Faculty of Health and Medical Sciences, University of Copenhagen, Copenhagen, Denmark.
93. Department of Medicine and Therapeutics, The Chinese University of Hong Kong, Hong Kong, China.
94. Li Ka Shing Institute of Health Sciences, The Chinese University of Hong Kong, Hong Kong, China.
95. Hong Kong Institute of Diabetes and Obesity, The Chinese University of Hong Kong, Hong Kong, China.
96. Estonian Genome Center, University of Tartu, Tartu, 50090, Estonia.
97. Centre for Fertility and Health, Norwegian Institute of Public Health, Oslo, N-0403, Norway.
98. Oxford NIHR Biomedical Research Centre, Oxford University Hospitals NHS Foundation Trust, John Radcliffe Hospital, Oxford, OX3 9DU, UK.
99. Centre for Genetic Origins of Health and Disease (GOHaD), The University of Western Australia, Crawley, WA, 6000, Australia.
100. Department of Medicine, Stanford School of Medicine, Stanford, CA 94305, USA.
101. Department of Genetics, University of North Carolina, Chapel Hill, NC 27599, USA.
102. Department of Clinical Epidemiology, Leiden University Medical Center, Leiden, 2333 ZA, The Netherlands.
103. Department of Public Health and Primary Care, Leiden University Medical Center, Leiden, 2333 ZA, The Netherlands.
104. Department of Public Health, Section of Epidemiology, Faculty of Health and Medical Sciences, University of Copenhagen, Copenhagen, Copenhagen, DK-1014, Denmark.
105. Department of Biostatistics, University of Liverpool, Liverpool, L69 3GL, UK.
106. Human Genetics Division, Cincinnati Children’s Hospital Medical Center, Cincinnati, OH 45229, USA.

107. Center for Prevention of Preterm Birth, Perinatal Institute, Cincinnati Children's Hospital Medical Center, Department of Pediatrics, University of Cincinnati College of Medicine, Cincinnati, OH 45229, USA.
108. March of Dimes Prematurity Research Center Ohio Collaborative, Cincinnati, OH 45229, USA.
109. Department of Pediatrics, University of Iowa, Iowa City, IA 52242, USA.
110. Department of Genetics and Bioinformatics, Norwegian Institute of Public Health, Oslo, N-0403, Norway.
111. Research Unit for Gynaecology and Obstetrics, Institute of Clinical Research, University of Southern Denmark, Odense, DK-5000, Denmark.
112. William Harvey Research Institute, Barts and the London School of Medicine and Dentistry, Queen Mary University of London, London, EC1M 6BQ, UK.
113. Medical Research Council (MRC), Social, Genetic and Developmental Psychiatry Centre, Institute of Psychiatry, Psychology & Neuroscience, King's College London, London, SE5 8AF, UK.
114. Division of Pediatric Endocrinology, Diabetes, and Metabolism, Department of Pediatrics, Columbia University Medical Center, New York, NY 10032, USA.
115. Obesity Prevention Program, Department of Population Medicine, Harvard Medical School and Harvard Pilgrim Health Care Institute, Boston, MA 02215, USA.
116. Department of Paediatrics, University of Cambridge, Cambridge, CB2 0QQ, UK.
117. Wellcome Sanger Institute, Hinxton, Cambridgeshire, CB10 1HH, UK.
118. School of Medicine and Public Health, Faculty of Medicine and Health, The University of Newcastle, Callaghan, NSW, 2308, Australia.
119. Research Centre of Applied and Preventive Cardiovascular Medicine, University of Turku, Turku, 20014, Finland.
120. Max Planck Institute for Psycholinguistics, Nijmegen, 6525 XD, The Netherlands.
121. Section of Genomics of Common Disease, Department of Medicine, Imperial College London, London, SW7 2AZ, UK.
122. Department of Clinical Physiology and Nuclear Medicine, Turku University Hospital, Turku, 20520, Finland.
123. BHF Centre for Cardiovascular Science, University of Edinburgh, Queen's Medical Research Institute, Edinburgh, EH16 4TJ, UK.
124. Department of Psychology, Mid Sweden University, Östersund, SE-831 25, Sweden.
125. Department of Medicine, Division of Endocrinology, Boston Children's Hospital, Boston, MA 02115, USA.
126. Program in Medical and Population Genetics, Broad Institute of Harvard and MIT, Cambridge, MA 02142, USA.
127. Center for Basic and Translational Obesity Research, Boston Children's Hospital, Boston, MA 02115, USA.
128. Saw Swee Hock School of Public Health, National University of Singapore, National University Health System, Singapore, 119077, Singapore.
129. Singapore Eye Research Institute, Singapore, 168751, Singapore.
130. Department of Genomics of Complexe Diseases, Imperial College, London, UK.
131. Department of Epidemiology, Cancer Center, University of Hawaii (Manoa), Honolulu, Hawaii, 96813, USA.
132. Department of Obstetrics and Gynecology, Erasmus MC, University Medical Center Rotterdam, Rotterdam, 3015 CE, The Netherlands.
133. Population Health Research Institute, St George's University of London, London, SW17 0RE, UK.
134. IMIM (Hospital del Mar Medical Research Institute), Barcelona, 08003, Spain.
135. Department of Statistics and Applied Probability, National University of Singapore, Singapore, 117546, Singapore.
136. Life Sciences Institute, National University of Singapore, Singapore, 117456, Singapore.

137. Division of Metabolic and Nutritional Medicine, Dr. von Hauner Children's Hospital, University of Munich Medical Center, Munich, 80337, Germany.
138. European Centre for Environment and Human Health, University of Exeter, Truro, TR1 3HD, UK.
139. Department of Internal Medicine, Erasmus MC, University Medical Center Rotterdam, Rotterdam, 3015 CE, The Netherlands.
140. Department of Public Health, Amsterdam Public Health Research Institute, Amsterdam UMC, location Academic Medical Center, University of Amsterdam, Amsterdam, 1105 AZ, The Netherlands.
141. Institute of Medical Statistics and Epidemiology, Technical University Munich, Munich, D-80333, Germany.
142. Institute of Medical Informatics, Biometry and Epidemiology, Ludwig Maximilians University, Munich, 81377, Germany.
143. Usher Institute for Population Health Sciences and Informatics, University of Edinburgh, Edinburgh, EH8 9AG, UK.
144. MRC Human Genetics Unit, Institute of Genetics and Molecular Medicine, University of Edinburgh, Edinburgh, EH4 2XU, UK.
145. Department of Clinical Epidemiology, Biostatistics and Bioinformatics, Amsterdam Public Health Research Institute, Amsterdam UMC, location Academic Medical Center, University of Amsterdam, Amsterdam, 1105 AZ, The Netherlands.
146. Institute of Translational Genomics, Helmholtz Zentrum München – German Research Center for Environmental Health, Neuherberg, Germany.
147. Division of Gastroenterology, Hepatology and Nutrition, The Children's Hospital of Philadelphia, Philadelphia, PA 19104, USA.

Supplementary Note 2. iPSYCH-BROAD Working Group

Collaborators in the iPSYCH-BROAD Working Group listed in alphabetical order.

Esben Agerbo[1,2,3], Jonas Bybjerg-Grauholm[1,4], Marie Bækved-Hansen[1,4], Mark J. Daly[5,6,7], Benjamin M. Neale[5,6,7], Carsten Bøcker Pedersen[1,2,3], Marianne Giørtz Pedersen[1,2,3].

1. iPSYCH, The Lundbeck Foundation Initiative for Integrative Psychiatric Research, Aarhus, Denmark.
2. National Centre for Register-Based Research, Aarhus University, Aarhus, Denmark.
3. Centre for Integrated Register-based Research, Aarhus University, Aarhus, Denmark.
4. Statens Serum Institut, Center for Neonatal Screening, Department for Congenital Disorders, Copenhagen, Denmark.
5. Analytic and Translational Genetics Unit, Department of Medicine, Massachusetts General Hospital and Harvard Medical School, Boston, USA.
6. Stanley Center for Psychiatric Research, Broad Institute of Harvard and MIT, Cambridge, Massachusetts, USA.
7. Program in Medical and Population Genetics, Broad Institute of Harvard and MIT, Cambridge, Massachusetts, USA.

Supplementary Note 3. Additional acknowledgements by study

ALSPAC: We are extremely grateful to all of the families who took part in this study, the midwives for their help in recruiting them, and the whole ALSPAC team, which includes interviewers, computer and laboratory technicians, clerical workers, research scientists, volunteers, receptionists, managers and nurses. Fetal GWAS data was generated by Sample Logistics and Genotyping Facilities at Wellcome Sanger Institute and LabCorp (Laboratory Corporation of America) using support from 23andMe.

BiB: Born in Bradford is only possible because of the enthusiasm and commitment of the Children and Parents in BiB. We are grateful to all the participants, practitioners and researchers who have made Born in Bradford happen. Fetal GWAS data was generated at the Bristol Bioresource Laboratories (University of Bristol).

CHOP: CHOP authors thank the network of primary care clinicians and the patients and families for their contribution to this project and to clinical research facilitated by the Pediatric Research Consortium (PeRC) at The Children's Hospital of Philadelphia. R. Chiavacci, E. Dabaghyan, A. (Hope) Thomas, K. Harden, A. Hill, C. Johnson-Honesty, C. Drummond, S. Harrison, F. Salley, C. Gibbons, K. Lilliston, C. Kim, E. Frackelton, G. Otieno, K. Thomas, C. Hou, K. Thomas and M.L. Garris provided expert assistance with genotyping and/or data collection and management. The authors would also like to thank S. Kristinsson, L.A. Hermannsson and A. Krisbjörnsson of Raförninn ehf for extensive software design and contributions.

COPSAC2000, COPSAC2010 and COPSAC-REGISTRY: We express our deepest gratitude to the children and families of the COPSAC2000, COPSAC2010 and COPSAC-REGISTRY cohort studies for all their support and commitment. We acknowledge and appreciate the unique efforts of the COPSAC research team.

DNBC and SSI-GE: We are very grateful to all DNBC and SSI-GE participants. We would also like to thank everyone involved in data collection and biological material handling.

GenR: The Generation R Study is conducted by the Erasmus Medical Center in close collaboration with the School of Law and Faculty of Social Sciences of the Erasmus University Rotterdam, the Municipal Health Service Rotterdam area, Rotterdam, the Rotterdam Homecare Foundation, Rotterdam and the Stichting Trombosedienst & Artsenlaboratorium Rijnmond [STAR-SHL], Rotterdam. We gratefully acknowledge the contribution of children and parents, general practitioners, hospitals, midwives and pharmacies in Rotterdam. The generation and management of GWAS genotype data for the Generation R Study was done at the Genetic Laboratory of the Department of Internal Medicine, Erasmus MC, the Netherlands. We would like to thank Karol Estrada, Dr. Tobias A. Knoch, Anis Abuseiris, Luc V. de Zeeuw, and Rob de Graaf, for their help in creating GRIMP, BigGRID, MediGRID, and Services@MediGRID/D-Grid, [funded by the German Bundesministerium fuer Forschung und Technology; grants 01 AK 803 A-H, 01 IG 07015 G] for access to their grid computing resources. We thank Pascal Arp, Mila Jhamai, Marijn Verkerk, Manoushka Ganesh, Lizbeth Herrera and Marjolein Peters for their help in creating, managing and QC of the GWAS database.

GPN: The analyses described in this manuscript included data obtained from the database of Genotype and Phenotype (dbGaP) found at http://www.ncbi.nlm.nih.gov/gap [accession number phs000714.v1.p1]. Samples and associated data were provided by the NICHD-funded Genomic and Proteomic Network for Preterm Birth Research (GPN-PBR). The contents of this report represent the views of the authors and do not necessarily represent the views of the NICHD or the GPN-PBR.

HAPO: Supported by grants from the Eunice Kennedy Shriver National Institute of Child Health and Human Development and the National Institute of Diabetes and Digestive and Kidney Diseases (R01-HD34242 and

R01-HD34243); the National Center for Research Resources (M01-RR00048 and M01-RR00080); and the American Diabetes Association; and grants to local field centers from Diabetes UK (RD04/0002756), Kaiser Permanente Medical Center, KK Women's and Children's Hospital, Mater Mother's Hospital, Novo Nordisk, the Myre Sim Fund of the Royal College of Physicians of Edinburgh, and the Howard and Carol Bernick Family Foundation.

INMA: INMA researchers would like to thank all the participants for their generous collaboration. A full roster of the INMA Project Investigators can be found at http://www.proyectoinma.org/presentacioninma/listado-investigadores/en_listado-investigadores.html.

MoBa_2008 and MoBa_HARVEST: The Norwegian Mother and Child Cohort Study (MoBa) is a prospective population-based pregnancy cohort study conducted by the Norwegian Institute of Public Health. Participants were recruited from all over Norway from 1999-2008. The women consented to participation in 41% of the pregnancies. The cohort now includes 114.500 children, 95.200 mothers and 75.200 fathers. Blood samples were obtained from both parents during pregnancy and from mothers and children (umbilical cord) at birth. We are grateful to all the participating families in Norway who take part in this on-going cohort study.

Raine Study: The authors are grateful to the Raine Study participants and their families, and to the Raine Study research staff for support they provided in cohort co-ordination and data collection over the last 29 years. The authors gratefully acknowledge the following institutes for providing funding for Core Management of the Raine Study: The University of Western Australia (UWA) , Curtin University, the Raine Medical Research Foundation, the UWA Faculty of Medicine, Dentistry and Health Sciences, the Telethon Kids Institute, the Women and Infants Research Foundation (King Edward Memorial Hospital), Murdoch University, The University of Notre Dame (Perth, Australia), and Edith Cowan University). The authors gratefully acknowledge the assistance of the Western Australian DNA Bank (National Health and Medical Research Council of Australia National Enabling Facility). This work was supported by resources provided by the Pawsey Supercomputing Centre with funding from the Australian Government and Government of Western Australia.

1958BC (WTCCC and DIL-T1DGC): This work made use of data and samples generated by the 1958 Birth Cohort (NCDS), which is managed by the Centre for Longitudinal Studies at the UCL Institute of Education. The authors are deeply grateful to the 1958 birth cohort participants for their longstanding commitment and support, and to all staff for cohort coordination and data collection.

Supplementary Note 4. Details of funding by study

ALSPAC: The work undertaken in this paper was funded by the ERC (Grant number 669545; DevelopObese), US National Institute of Diabetes and Digestive and Kidney Diseases (R01 DK10324) and Wellcome (WT088806), with the WT088806 grant also providing funds for completion of genome wide genotyping on the ALSPAC mothers. ALSPAC offspring (fetal) GWAS data was generated by Sample Logistics and Genotyping Facilities at the Wellcome Sanger Institute and LabCorp (Laboratory Corporation of America) using support from 23andMe. Additional maternal data were funded by the British Heart Foundation (SP/07/008/24066) and Wellcome (WT087997). The UK Medical Research Council and Wellcome (Grant ref: 102215/2/13/2) and the University of Bristol provide core support for ALSPAC.

BiB: BiB receives core funding from Wellcome (WT101597MA), a joint grant from the UK Medical Research Council (MRC) and Economic and Social Science Research Council (ESRC) (MR/N024397/1) and from the British Heart Foundation (CS/16/4/32482). Specific funding for this study (including for maternal and offspring genotyping) come from the US NIH (DK10324), the European Research Council (Grant: DevelopObese; 669545) and UK Medical Research Council (MC_UU_12013/5).

CHOP: CHOP was financially supported by an Institute Development Award from the Children's Hospital of Philadelphia, a Research Development Award from the Cotswold Foundation, NIH grant R01 HD056465 and the Daniel B. Burke Endowed Chair for Diabetes Research (SFAG).

COPSAC2000, COPSAC2010 and COPSAC-REGISTRY: All funding received by COPSAC is listed on www.copsac.com. The Lundbeck Foundation (Grant no R16-A1694); The Ministry of Health (Grant no 903516); Danish Council for Strategic Research (Grant no 0603-00280B) and The Capital Region Research Foundation have provided core support to the COPSAC research center.

DNBC: The Danish National Birth Cohort (DNBC) is a result of major grants from the Danish National Research Foundation, the Danish Pharmacists' Fund, the Egmont Foundation, the March of Dimes Birth Defects Foundation, the Augustinus Foundation, and the Health Fund of the Danish Health Insurance Societies. The DNBC biobank is a part of the Danish National Biobank resource, which is supported by the Novo Nordisk Foundation. The generation of GWAS genotype data for the DNBC samples was carried out within the Gene Environment Association Studies (GENEVA) Consortium with funding provided through the National Institutes of Health's Genes, Environment, and Health Initiative (U01HG004423; U01HG004446; U01HG004438).

EFSOCH: The Exeter Family Study of Childhood Health (EFSOCH) was supported by South West NHS Research and Development, Exeter NHS Research and Development, the Darlington Trust and the Peninsula National Institute of Health Research (NIHR) Clinical Research Facility at the University of Exeter. The opinions given in this paper do not necessarily represent those of NIHR, the NHS or the Department of Health. Genotyping of the EFSOCH study samples was funded by the Welcome Trust and Royal Society grant WT104150.

GenR: The general design of Generation R Study is made possible by financial support from the Erasmus Medical Center, Rotterdam, the Erasmus University Rotterdam, the Netherlands Organization for Health Research and Development (ZonMw), the Netherlands Organisation for Scientific Research (NWO), the Ministry of Health, Welfare and Sport and the Ministry of Youth and Families. Fernando Rivadeneira received additional funding from the Netherlands Organization for Health Research and Development (VIDI 016.136.367). This project received funding from the European Union's Horizon 2020 research and innovation programme under grant agreements 733206 (LIFECYCLE) and 633595 (DynaHEALTH). Vincent Jaddoe received additional funding from the Netherlands Organization for Health Research and

Development (VIDI 016.136.361) and the European Research Council (ERC Consolidator Grant, ERC-2014-CoG-648916).

HAPO: This study was supported by National Institutes of Health (NIH) grants HD-34242, HD-34243, HG-004415, and CA-141688, Institutes of Health Research–INMD (Funding Reference Number 110791), and by the American Diabetes Association.

INMA: This study was funded by grants from Instituto de Salud Carlos III (Red INMA G03/176 and CB06/02/0041), FIS-FEDER 03/1615, 04/1509, 04/1112, 04/1931, 05/1079, 05/1052, 06/1213, 07/0314, 09/02647, 11/01007, 11/02591, 13/02032, 13/1944, PI041436, PI081151, 11/00178, 97/0588, 00/0021-2, PI061756, PS0901958, 14/00891, and 14/01687, Spanish Ministry of Science and Innovation (SAF2008-00357), European Commission (ENGAGE project and grant agreement HEALTH-F4-2007-201413), Fundació La Marató de TV3, Generalitat de Catalunya-CIRIT 1999SGR 00241 and Conselleria de Sanitat Generalitat Valenciana. Part of the DNA extractions and genotyping was performed at the Spanish National Genotyping Centre (CEGEN-Barcelona).

iPSYCH: This study was funded by The Lundbeck Foundation, Denmark. This research has been conducted using the Danish National Biobank resource supported by the Novo Nordisk Foundation. Grant numbers: R248-2017-2003-Period III: 1 March 2018 -28 February 2021; R155-2014-1724: Period II: 1 March 2015 –28 Februar 2018 and R102-A9118: Period I: 1 March 2012 –28 February 2015. Genotyping of iPSYCH samples was further supported by grants from the Stanley Foundation, the Simons Foundation (SFARI 311789), and the NIMH (5U01MH094432-02).

MoBa_2008 and MoBa_HARVEST: The Norwegian Mother and Child Cohort Study is supported by the Norwegian Ministry of Health and Care Services and the Ministry of Education and Research, NIH/NINDS (grant no.1 UO1 NS 047537-01 and grant no.2 UO1 NS 047537-06A1). The analyses were supported by the Research Council of Norway (grant FRIMEDBIO ES547711 to Bo Jacobsson), the March of Dimes Foundation Prematurity Research Initiative Program (grant 21-FY16-121 to Bo Jacobsson), and the European Research Council (Advanced Grant, no. 293574 to Pål R. Njølstad).

NFBC1966 and NFBC1986: NFBC1966 received financial support from the Academy of Finland (project grants 104781, 120315, 129269, 1114194, 24300796, Center of Excellence in Complex Disease Genetics and SALVE), University Hospital Oulu, Biocenter, University of Oulu, Finland (75617), NHLBI grant 5R01HL087679-02 through the STAMPEED program (1RL1MH083268-01), NIH/NIMH (5R01MH63706:02), ENGAGE project and grant agreement HEALTH-F4-2007-201413, EU FP7 EurHEALTHAgeing -277849, the Medical Research Council, UK (G0500539, G0600705, G1002319, PrevMetSyn/SALVE) and the MRC, Centenary Early Career Award. The program is currently being funded by the H2020-633595 DynaHEALTH action, academy of Finland EGEA-project (285547) and EU H2020 ALEC project (Grant Agreement 633212). NFBC1986 received financial support from EU QLG1-CT-2000-01643 (EUROBLCS) Grant no. E51560, NorFA Grant no. 731, 20056, 30167, USA / NIHH 2000 G DF682 Grant no. 50945.

Raine Study: This study was supported by the National Health and Medical Research Council of Australia [grant numbers 572613 and 403981] and the Canadian Institutes of Health Research [grant number MOP-82893].

SSI-GE: The Statens Serum Institut Genetic Epidemiology (SSI-GE) cohort is comprised of samples with GWAS data generated in previous research projects conducted at Statens Serum Institut. Samples for these projects were drawn from the Danish National Biobank, which is supported by the Novo Nordisk Foundation with additional partial support from the Lundbeck Foundation and the Danish Medical Research Council.

STRIP: This work was supported by Academy of Finland [grant numbers: 206374, 294834, 251360, 275595]; Juho Vainio Foundation; Finnish Cultural Foundation; Finnish Foundation for Cardiovascular Research; Sigrid Juselius Foundation; Yrjö Jahnsson Foundation; Finnish Diabetes Research Foundation; Novo Nordisk Foundation; Strategic Research Funding from the University of Oulu, Finnish Ministry of Education and Culture; Special Governmental Grants for Health Sciences Research, Turku University Hospital; and Turku University Foundation.

1958BC (DIL-T1DGC and WTCCC): The management of the 1958 Birth Cohort is funded by the Economic and Social Research Council (grant number ES/M001660/1). Access to these resources was enabled via the 58READIE Project funded by Wellcome and Medical Research Council (grant numbers WT095219MA and G1001799). DNA collection was funded by MRC grant G0000934 and cell-line creation by Wellcome grant 068545/Z/02. DIL-T1DGC used resources provided by the Type 1 Diabetes Genetics Consortium, a collaborative clinical study sponsored by the National Institute of Diabetes and Digestive and Kidney Diseases (NIDDK), National Institute of Allergy and Infectious Diseases, National Human Genome Research Institute, National Institute of Child Health and Human Development, and Juvenile Diabetes Research Foundation International (JDRF) and supported by U01 DK062418. The 1958BC (WTCCC) makes use of data generated by the Wellcome Trust Case-Control Consortium. A full list of investigators who contributed to generation of the data is available from the Wellcome Trust Case-Control Consortium website. Funding for the project was provided by the Wellcome Trust under the award 076113. Great Ormond Street Hospital/University College London, Institute of Child Health receives a proportion of funding from the Department of Health's National Institute for Health Research (NIHR) ('Biomedical Research Centres' funding).
