# Supplementary material for: Variants in the fetal genome near pro-inflammatory cytokine genes on 2q13 associate with gestational duration
Source: Nat Commun. 2019 Sep 2;10:3927. doi: 10.1038/s41467-019-11881-8 (PMC6718389; doi:10.1038/s41467-019-11881-8)
Supplement: Supplementary file 3 — Description of Additional Supplementary Files [file 41467_2019_11881_MOESM3_ESM.pdf]

Description of Supplementary Data files

Supplementary Data 1. Sample characteristics of the discovery and replication study cohorts.

Supplementary Data 2. Annotated association results for gestational duration and postterm birth for all variants at the 2q13 locus with $P < 1 \times 10^{-4}$ in the discovery stage. The table is sorted by discovery stage *P* value and shows marker name and base pair position (NCBI build 37); effect and alternative allele; effect allele frequency; direction of effect in each contributing cohort; discovery stage *P* value; heterogeneity test results; effect estimate with 95% confidence interval; squared Pearson correlation coefficient (r^2) of imputed SNP allele dosage to allele dosage for the top SNP at the locus; eQTL results from the GTEx and GEUVADIS consortia; function class and name of nearest gene; GWAS catalog annotation; enhancer information from the GeneHancer database. In the eQTLs columns, +1 or -1 behind the gene name indicates whether the effect allele of the SNP corresponds to increased or decreased expression of the gene.

Supplementary Data 3. Ensembl Variant Effect Predictor (VEP) annotation of variants at the 2q13 locus with $P < 1 \times 10^{-4}$ in the discovery stage analyses of gestational duration and postterm birth. Each variant may have more than one line in the table, if it affects multiple transcripts. The table is sorted by base pair position and shows the variant allele used to calculate the consequence; Ensembl stable ID of affected gene; Ensembl stable ID of feature; type of feature (Transcript, RegulatoryFeature, MotifFeature); Consequence; cDNA_position (relative position in cDNA sequence); CDS_position (relative position of base pair in coding sequence); Protein_position (relative position of amino acid in protein); Amino acids; Codons (the alternative codons with the variant base in upper case); Existing variation (known identifier of existing variant); Extra information including SIFT and Polyphen scores, as well as global allele frequencies.

Supplementary Data 4. Gestational duration association analysis of exome sequencing data. The first tab contains results of single variant association tests for variants in chr2:113MB:114MB with $P < 0.01$. CHR:POS denotes chromosomal position on GRCh37/hg19. A1 and A0 denote effect and non-effect allele, respectively. MAF is minor allele frequency, AC is the allele count for the minor allele, while R2 is the linkage disequilibrium r^2 to the lead variant rs7594852. For each variant effect size estimates (BETA), standard error (SE) and *P* value for association with quantile transformed gestational duration are given as well as corresponding results when conditioning on the lead variant rs7594852 in the association analysis (BETA_COND, SE_COND and P_COND). The second tab contains results of gene-based rare-variants association tests for genes within chr2:113MB:114MB using test SKAT-O approach. CHR, START and END denote chromosomal position on GRCh37/hg19. The fraction of individuals with rare variants (FRAC_WITH_RARE), the number of variants (NUM_ALL_VARS), the number of variants after data cleaning (NUM_PASS_VARS) and the number of singleton variants (NUM_SINGLE_VARS) in each gene are given, along with the SKAT-O *P* value and the optimal weight (STATRHO) for the test.

Supplementary Data 5. Association analyses for four autosomal variants previously reported in a maternal GWAS of gestational duration and preterm birth. The first tab contains fetal results for quantile transformed gestational duration (discovery meta-analysis) and gestational duration in days (based on 51,357 infants from the iPSYCH study), as well as maternal results for gestational duration in days from the published maternal GWAS (PMID: 28877031). The second tab contains results of joint analyses in 15,588 mother-child pairs for the same four variants.

Supplementary Data 6. Results of genetic correlation analyses between the discovery stage results for gestational duration and 690 traits and diseases in LDHub. The table shows trait; PubMed ID (if applicable); trait category (ukbb = UK Biobank); genetic correlation (r_g); standard error of r_g, z statistic for test of H_0: r_g = 0 with corresponding *P* value; observed scale heritability of the trait (h2_obs) and its standard error; single-trait LD Score regression intercept (h2_int) for the trait and its standard error; cross-trait LD Score regression intercept (gcov_int) and its standard error. All traits were analyzed in individuals of European ancestry and SNPs from the MHC region (chromosome 6 26M~34M) were not included. Genetic correlations that were significant after Bonferroni correction are shown on light blue background. The first tab contains results for quantile transformed gestational duration, the second tab contains results for fetal effect (gestational age in days) adjusted for the maternal genotype using the weighted linear model (WLM) approach (PMID: 31043758), and the third tab contains results for maternal effect (gestational age in days) adjusted for fetal genotype using the WLM approach.

Supplementary Data 7. Predicted regulatory function of variants at the 2q13 locus in relevant cell types. Variants are ranked according to the total number of functional genomics datasets that they intersect. The table shows marker name and position (NCBI build 37); number of dataset that the variant intersects; cell type; annotation track; start and end of the track; track label.
