# Supplementary material for: Variants in the fetal genome near pro-inflammatory cytokine genes on 2q13 associate with gestational duration
Source: Nat Commun. 2019 Sep 2;10:3927. doi: 10.1038/s41467-019-11881-8 (PMC6718389; doi:10.1038/s41467-019-11881-8)
Supplement: Supplementary file 11 — Reporting Summary [file 41467_2019_11881_MOESM11_ESM.pdf]

nature research

Corresponding author(s): Bjarke Feenstra

Reporting Summary

Nature Research wishes to improve the reproducibility of the work that we publish. This form provides structure for consistency and transparency in reporting. For further information on Nature Research policies, see Authors & Referees and the Editorial Policy Checklist.

Statistical parameters

When statistical analyses are reported, confirm that the following items are present in the relevant location (e.g. figure legend, table legend, main text, or Methods section).

n/a	Confirmed	
☐	☒	The exact sample size (n) for each experimental group/condition, given as a discrete number and unit of measurement
☐	☒	An indication of whether measurements were taken from distinct samples or whether the same sample was measured repeatedly
☐	☒	The statistical test(s) used AND whether they are one- or two-sided<br>*Only common tests should be described solely by name; describe more complex techniques in the Methods section.*
☐	☒	A description of all covariates tested
☐	☒	A description of any assumptions or corrections, such as tests of normality and adjustment for multiple comparisons
☐	☒	A full description of the statistics including central tendency (e.g. means) or other basic estimates (e.g. regression coefficient) AND variation (e.g. standard deviation) or associated estimates of uncertainty (e.g. confidence intervals)
☐	☒	For null hypothesis testing, the test statistic (e.g. F, t, r) with confidence intervals, effect sizes, degrees of freedom and P value noted<br>*Give P values as exact values whenever suitable.*
☒	☐	For Bayesian analysis, information on the choice of priors and Markov chain Monte Carlo settings
☒	☐	For hierarchical and complex designs, identification of the appropriate level for tests and full reporting of outcomes
☐	☒	Estimates of effect sizes (e.g. Cohen's d, Pearson's r), indicating how they were calculated
☐	☒	Clearly defined error bars<br>*State explicitly what error bars represent (e.g. SD, SE, CI)*

Our web collection on statistics for biologists may be useful.

Software and code

Policy information about availability of computer code

Data collection

The genotype data in the contributing studies were generated using GWAS SNP arrays with genotype calling performed as described in Supplementary Table 1. Genotyping in the Iowa cohort was done using TaqMan assays as described in the Methods section.

The GPN study genotype and phenotype data were downloaded from dbGaP (https://www.ncbi.nlm.nih.gov/gap, accession number: phs000714.v1.p1).

Imputation was done separately for contributing studies using publicly available data from the Haplotype Reference Consortium (http://www.haplotype-reference-consortium.org) or the 1000 Genomes Project (http://www.1000genomes.org/).

Annotation was done centrally after meta-analysis using ANNOVAR (version 2017Jun01; http://annovar.openbioinformatics.org/) and Ensembl VEP (version 94 (GRCh37); https://www.ensembl.org/vep) based on publicly available data as described in the Methods.

Computational prediction of gene regulatory mechanisms was done using Cis-BP (build 1.02; http://cisbp.ccbr.utoronto.ca/) based on publicly available data as described in the Methods.

Data analysis

Association analysis: done separately for contributing studies using PLINK (https://www.cog-301 genomics.org/plink2), SNPTEST (https://mathgen.stats.ox.ac.uk/genetics_software/snptest/snptest.html), or RVTESTS (https://github.com/zhanxw/rvtests)

Meta-analysis: METAL (version 2011-03-25; http://www.sph.umich.edu/csg/abecasis/metal/)

LD Score regression: LDSC (version 1.0.0; https://github.com/bulik/ldsc)

Power analysis and other additional statistical analyses: R (version 3.5.0; https://www.r-project.org/)

For manuscripts utilizing custom algorithms or software that are central to the research but not yet described in published literature, software must be made available to editors/reviewers upon request. We strongly encourage code deposition in a community repository (e.g. GitHub). See the Nature Research guidelines for submitting code & software for further information.

Data

Policy information about availability of data

All manuscripts must include a data availability statement. This statement should provide the following information, where applicable:

- Accession codes, unique identifiers, or web links for publicly available datasets
- A list of figures that have associated raw data
- A description of any restrictions on data availability

The GWAS summary statistics from the meta-analyses of all four outcomes (early preterm birth, preterm birth, postterm birth, and gestational duration) will be made publicly available on the EGG Consortium website (http://egg-consortium.org/) upon publication of the article as has been done in previous EGG Consortium studies. These summary statistics include the source data underlying Figure 1 and Supplementary Figures 2, 3, 4, 6 and 7. The source data underlying Figures 2 and 3 and Supplementary Figures 5 and 8–11 are provided as a Source Data File.

Field-specific reporting

Please select the best fit for your research. If you are not sure, read the appropriate sections before making your selection.

☒ Life sciences ☐ Behavioural & social sciences ☐ Ecological, evolutionary & environmental sciences

For a reference copy of the document with all sections, see nature.com/authors/policies/ReportingSummary-flat.pdf

Life sciences study design

All studies must disclose on these points even when the disclosure is negative.

Sample size	All eligible studies with gestational duration information available (that we were aware of) were invited to participate in the meta-analysis. Our statistical power calculations indicate that most of our association analyses were well-powered. See Supplementary Figure 10 and Supplementary Table 1 for more information.
Data exclusions	We excluded pregnancies based on the following pre-established criteria: 1) stillbirths; 2) twins or any multiple births; 3) ancestry outliers using principal component analysis; 4) outliers in birth weight or birth length (gestational duration possibly wrong); 5) Caesarian section, if due to pregnancy complications; Caesarian sections due to complications during labor were not excluded. Caesarian sections were allowed for cases in the postterm birth analysis; 6) Physician initiated births (induced births were allowed for cases in the postterm birth analysis); 7) placental abruption, placenta previa, pre-eclampsia/eclampsia, hydramnios, placental insufficiency, cervical insufficiency, isoimmunization, gestational diabetes, cervical cerclage; 8) pre-existing medical conditions in the mother, such as diabetes, hypertension, autoimmune diseases (including systemic lupus erythematosus, rheumatoid arthritis and sclerodermia), immuno-compromised patients; and 9) known congenital anomalies. Further, the study sample was restricted to individuals of European ancestry, in most cohorts by principal component analysis. Some cohorts were not able to perform exclusions according to all criteria, but applied as many criteria as possible (see Supplementary Data 1 for details).<br><br>Our exclusion criteria aimed to focus on ‘natural’ gestational duration rather than specific causes such as preterm birth due to pregnancy complications, assisted delivery or congenital anomalies.
Replication	The association of the lead SNP at the 2q13 locus with gestational duration was replicated in 9291 additional infants (replication P = 0.00369; combined P=3.96e-14).<br><br>The association of the same variant with postterm birth did not reach nominal significance in the replication set of 670 cases and 5626 controls. For the postterm birth replication analysis, statistical power was modest at 40%. However, the direction of effects was consistent with the discovery stage result.<br><br>The two loci that were associated with early preterm birth at P<5e-8 did not replicate in the replication set of 107 cases and 865 controls. These loci also did not replicate in a set of 276 early preterm birth mother-father-child trios.
Randomization	No randomization was performed, since existing studies with gestational duration information available were analyzed. These studies included population-based cohorts and case-control studies. For the latter, association analyses of the four outcomes of interest were done in strata defined by disease group to avoid false-positive associations driven by genetic susceptibility to the disease in question.
Blinding	Not relevant, since existing studies with gestational duration information available were analyzed.

Reporting for specific materials, systems and methods

Materials & experimental systems

n/a	Involved in the study	
☒	☐	Unique biological materials
☒	☐	Antibodies
☒	☐	Eukaryotic cell lines
☒	☐	Palaeontology
☒	☐	Animals and other organisms
☐	☒	Human research participants

Methods

n/a	Involved in the study	
☒	☐	ChIP-seq
☒	☐	Flow cytometry
☒	☐	MRI-based neuroimaging

Human research participants

Policy information about studies involving human research participants

Population characteristics	This is described in detail for each of the participating studies in Supplementary Table 1.
Recruitment	The meta-analysis includes population-based cohorts, as well as case-control studies of various diseases. For the latter, association analyses of the four outcomes of interest were done in strata defined by disease group to avoid false-positive associations driven by genetic susceptibility to the disease in question. It is reassuring that we we did not observe heterogeneity of effect estimates between studies of various design for the 2q13 locus.
